# Geographic Distribution of *Staphylococcus aureus* Causing Invasive Infections in Europe: A Molecular-Epidemiological Analysis

**DOI:** 10.1371/journal.pmed.1000215

**Published:** 2010-01-12

**Authors:** Hajo Grundmann, David M. Aanensen, Cees C. van den Wijngaard, Brian G. Spratt, Dag Harmsen, Alexander W. Friedrich, the European Staphylococcal Reference Laboratory Working Group

**Affiliations:** 1 National Institute for Public Health and the Environment, Bilthoven, The Netherlands; 2 Department of Medical Microbiology, University Medical Centre, Groningen, The Netherlands; 3 Department of Infectious Disease Epidemiology, Imperial College London, London, United Kingdom; 4 Department of Periodontology, University Hospital Münster, Germany; 5 Institute of Hygiene, University Hospital Münster, Germany; University of California San Francisco and San Francisco General Hospital, United States of America

## Abstract

**Background:**

*Staphylococcus aureus* is one of the most important human pathogens and methicillin-resistant variants (MRSAs) are a major cause of hospital and community-acquired infection. We aimed to map the geographic distribution of the dominant clones that cause invasive infections in Europe.

**Methods and Findings:**

In each country, staphylococcal reference laboratories secured the participation of a sufficient number of hospital laboratories to achieve national geo-demographic representation. Participating laboratories collected successive methicillin-susceptible (MSSA) and MRSA isolates from patients with invasive *S. aureus* infection using an agreed protocol. All isolates were sent to the respective national reference laboratories and characterised by quality-controlled sequence typing of the variable region of the staphylococcal *spa* gene (*spa* typing), and data were uploaded to a central database. Relevant genetic and phenotypic information was assembled for interactive interrogation by a purpose-built Web-based mapping application. Between September 2006 and February 2007, 357 laboratories serving 450 hospitals in 26 countries collected 2,890 MSSA and MRSA isolates from patients with invasive *S. aureus* infection. A wide geographical distribution of *spa* types was found with some prevalent in all European countries. MSSA were more diverse than MRSA. Genetic diversity of MRSA differed considerably between countries with dominant MRSA *spa* types forming distinctive geographical clusters. We provide evidence that a network approach consisting of decentralised typing and visualisation of aggregated data using an interactive mapping tool can provide important information on the dynamics of MRSA populations such as early signalling of emerging strains, cross border spread, and importation by travel.

**Conclusions:**

In contrast to MSSA, MRSA *spa* types have a predominantly regional distribution in Europe. This finding is indicative of the selection and spread of a limited number of clones within health care networks, suggesting that control efforts aimed at interrupting the spread within and between health care institutions may not only be feasible but ultimately successful and should therefore be strongly encouraged.

*Please see later in the article for the Editors' Summary*

## Abstract 2

Hajo Grundmann and colleagues describe the development of a new interactive mapping tool for analyzing the spatial distribution of invasive *Staphylococcus aureus* clones.

## Editors' Summary

**Background:**

The bacterium *Staphylococcus aureus* lives on the skin and in the nose of about a third of healthy people. Although *S. aureus* usually coexists peacefully with its human carriers, it is also an important disease-causing organism or pathogen. If it enters the body through a cut or during a surgical procedure, *S. aureus* can cause minor infections such as pimples and boils or more serious, life-threatening infections such as blood poisoning and pneumonia. Minor *S. aureus* infections can be treated without antibiotics—by draining a boil, for example. Invasive infections are usually treated with antibiotics. Unfortunately, many of the *S. aureus* clones (groups of bacteria that are all genetically related and descended from a single, common ancestor) that are now circulating are resistant to methicillin and several other antibiotics. Invasive methicillin-resistant *S. aureus* (MRSA) infections are a particular problem in hospitals and other health care facilities (so-called hospital-acquired MRSA infections), but they can also occur in otherwise healthy people who have not been admitted to a hospital (community-acquired MRSA infections).

**Why Was This Study Done?:**

The severity and outcome of an *S. aureus* infection in an individual depends in part on the ability of the bacterial clone with which the individual is infected to cause disease—the clone's “virulence.” Public-health officials and infectious disease experts would like to know the geographic distribution of the virulent *S. aureus* clones that cause invasive infections, because this information should help them understand how these pathogens spread and thus how to control them. Different clones of *S. aureus* can be distinguished by “molecular typing,” the determination of clone-specific sequences of nucleotides in variable regions of the bacterial genome (the bacterium's blueprint; genomes consist of DNA, long chains of nucleotides). In this study, the researchers use molecular typing to map the geographic distribution of MRSA and methicillin-sensitive *S. aureus* (MSSA) clones causing invasive infections in Europe; a MRSA clone emerges when an MSSA clone acquires antibiotic resistance from another type of bacteria so it is useful to understand the geographic distribution of both MRSA and MSSA.

**What Did the Researchers Do and Find?:**

Between September 2006 and February 2007, 357 laboratories serving 450 hospitals in 26 European countries collected almost 3,000 MRSA and MSSA isolates from patients with invasive *S. aureus* infections. The isolates were sent to the relevant national staphylococcal reference laboratory (SRL) where they were characterized by quality-controlled sequence typing of the variable region of a staphylococcal gene called *spa* (*spa* typing). The *spa* typing data were entered into a central database and then analyzed by a public, purpose-built Web-based mapping tool (SRL-Maps), which provides interactive access and easy-to-understand illustrations of the geographical distribution of *S. aureus* clones. Using this mapping tool, the researchers found that there was a wide geographical distribution of *spa* types across Europe with some types being common in all European countries. MSSA isolates were more diverse than MRSA isolates and the genetic diversity (variability) of MRSA differed considerably between countries. Most importantly, major MRSA *spa* types occurred in distinct geographical clusters.

**What Do These Findings Mean?:**

These findings provide the first representative snapshot of the genetic population structure of *S. aureus* across Europe. Because the researchers used *spa* typing, which analyzes only a small region of one gene, and characterized only 3,000 isolates, analysis of other parts of the *S. aureus* genome in more isolates is now needed to build a complete portrait of the geographical abundance of the *S. aureus* clones that cause invasive infections in Europe. However, the finding that MRSA *spa* types occur mainly in geographical clusters has important implications for the control of MRSA, because it indicates that a limited number of clones are spreading within health care networks, which means that MRSA is mainly spread by patients who are repeatedly admitted to different hospitals. Control efforts aimed at interrupting this spread within and between health care institutions may be feasible and ultimately successful, suggest the researchers, and should be strongly encouraged. In addition, this study shows how, by sharing typing results on a Web-based platform, an international surveillance network can provide clinicians and infection control teams with crucial information about the dynamics of pathogens such as *S. aureus*, including early warnings about emerging virulent clones.

**Additional Information:**

Please access these Web sites via the online version of this summary at http://dx.doi.org/10.1371/journal.pmed.1000215.

- This study is further discussed in a *PLoS Medicine* Perspective by Franklin D. Lowy
- The UK Health Protection Agency provides information about *Staphylococcus aureus*
- The UK National Health Service Choices Web site has pages on staphylococcal infections and on MRSA
- The US National Institute of Allergy and Infectious Disease has information about MRSA
- The US Centers for Disease Control and Infection provides information about MRSA for the public and professionals
- MedlinePlus provides links to further resources on staphylococcal infections and on MRSA (in English and Spanish)
- SRL-Maps can be freely accessed

## Introduction

*Staphylococcus aureus* is the main cause of purulent infection in humans [1]. *S. aureus* has the potential for local as well as disseminated infection and can cause lesions in all tissues and anatomical sites. Infections can be either acquired in the community or in association with health care. The position of *S. aureus* as one of the most important human pathogens is largely due to its virulence potential and ubiquitous occurrence as a coloniser in humans, domestic animals, and livestock [2]. Between 25% and 35% of healthy human individuals carry *S. aureus* on the skin or mucous membranes [3]. Any injury that compromises epithelial integrity, trauma, medical or surgical interventions, as well as viral infections, can lead to tissue invasion [4]–[6]. It is assumed that severity and outcome depend largely on the virulence of the introduced strain and the immune repertoire of the host [7],[8]. Occasionally, *S. aureus* acquires enhanced virulence and antimicrobial resistance through horizontal DNA transfer and maintains these mobile genetic elements in a predominantly clonal genomic background. Thus, clones of *S. aureus* are relatively stable and mainly diversify by the accumulation of single nucleotide substitutions in the absence of frequent interstrain recombination. It is therefore possible to discern different clones and clonal lineages by molecular typing [9]. This method allows several important observations to be made regarding the evolution, epidemiology, and spread of clones with particular public health importance, such as hospital-, community-, and livestock-associated methicillin-resistant *S. aureus* (MRSA). For MRSA, this surveillance is particularly important because it appears that certain clones have disseminated over wide geographical regions and are threatening major improvements in curative and public health medicine [10]. A geographically detailed description of this expansion on a continent-wide scale has been inadequate, however, due to the lack of appropriate surveys and agreement on a consistent application of standardized molecular typing approaches. At the same time, little is known about the population structure and geographical abundance of methicillin-susceptible *S. aureus* (MSSA), which provides the genetic reservoir from which MRSA emerge.

The present study was designed to fill these knowledge gaps and to provide (i) the first representative and contemporaneous snapshot of the genetic population structure of *S. aureus* (based on *spa* typing) that cause invasive infection in the European region; (ii) insights into the geographic distribution of different clones among MSSA and MRSA on a continent-wide scale; and (iii) a public Web-based mapping tool supplying interactive access and an intuitive illustration of the results generated by this large-scale typing initiative. The current study was also set up to establish the logistics for future collaborative studies that will continue to improve crucial knowledge for clinicians and diagnostic laboratories about the geographic and temporal dynamics of the MSSA/MRSA clones and their epidemic patterns in neighbouring geographical areas. Lastly, the study is intended to additionally strengthen the role of the *S. aureus* Reference Laboratories (SRLs) by exposing and communicating potentially important public health threats to health professionals and the general public.

## Methods

### *spa* Typing

Epidemiological typing uses highly discriminatory genetic markers that characterize human pathogens allowing the identification of isolates that are closely related due to recent common ancestry. The *spa* locus of *S. aureus* codes for protein A, a species-specific gene product known for its IgG binding capacity. This locus is highly polymorphic due to an internal variable region of short tandem repeats, which vary not only in numbers but also because of nucleotide substitutions within individual repeat units [11]. DNA sequences of the *spa* gene therefore provide portable, unambiguous, and biologically meaningful molecular typing data, which have demonstrated their utility for epidemiological purposes such as transmission and outbreak investigations at various geographical levels [12],[13].

### Capacity Building

During three workshops organised for technical personnel from 28 European SRLs, participants received hands-on training in *spa* sequence typing and *spa* sequence analysis according to a standard protocol using a purpose-designed software tool, StaphType, developed by Ridom GmbH [13]. Proficiency testing was carried out by mailing each SRL five well-characterized *S. aureus* isolates and five sequence chromatograms (trace files) of known *spa* types as described previously [14],[15]. All laboratories participating in the structured survey described here fulfilled quantifiable quality criteria, which consisted of an unambiguous base-calling of all sequenced nucleotides for both forward and reverse sequencing runs of the test panel.

### Structured Survey

A protocol was drawn up and circulated for comment to all SRLs and agreed upon in April 2006. Following this protocol, European SRLs were asked to identify approximately 20 laboratories that serve hospitals and that are geographically and demographically representative of their country, and secure their participation. These laboratories were chosen from those that regularly participate in the European Antimicrobial Resistance Surveillance System (EARSS). For 6 mo, from September 2006 until February 2007, these participants were asked to submit the first five successive MSSA isolates and the first five successive MRSA isolates from individual patients with invasive infection. An invasive infection was defined as a localised or systemic inflammatory response to the presence of *S. aureus* at otherwise sterile anatomical sites. Isolates were dispatched by the participating laboratories to the SRLs accompanied by additional information, including the EARSS laboratory identifier, the sample number, the date of isolation, origin of clinical specimen, demographic detail (such as age and gender), epidemiological context (hospital-acquired when symptoms developed more than 48 h after admission or as community-onset otherwise), isoxazolylpenicillin- (i.e., oxacillin) or cefoxitin-resistance, and all-cause mortality 14 d after isolation of the first invasive isolate. SRLs confirmed MRSA by *mec*A PCR or determination of minimum inhibitory concentration for oxacillin together with PBP2a agglutination. Additional data were uploaded to the database and Web application if available. These consisted of staphylococcal cassette chromosome *mec* (SCC*mec*) typing, and identification of *luk*-PV genes, indicative of Panton-Valentine Leukocidin (PVL). All SRLs preserved the isolates in strain collections and performed *spa* sequence typing according to the standard protocol, uploaded the sequence information, and made this available by synchronisation with the central Ridom SpaServer (www.spaserver.ridom.de) curated by SeqNet.org at the Institute of Hygiene, University Hospital Münster, Germany [15],[16]. Submitted sequences were quality controlled by comparison with accompanying chromatograms (trace files) and excluded if stringent quality criteria for excellent sequencing data were not fulfilled. *spa* types were grouped into *spa* complexes if a single genetic event such as single insertions, single deletions, or single nucleotide polymorphism could account for the observed sequence divergence. In the following the designation of *spa* types indicated by lower case “t” follow the nomenclature used by the *spa* server and multilocus sequence types are reported as sequence type (ST) according to convention [17]. Finally, the SCC*mec* type is also added to a string consisting of *spa* type/ST/SCCmec all in parenthesis, e.g., (t032/ST22/SCC*mec*IV).

Epidemiological and typing data were communicated in parallel to a central SQL database at the National Institute for Public Health and the Environment (RIVM) of the Netherlands. For each participating laboratory, SRLs also provided the postal address and indicated their administrative region (such as region, province, state, department, or NUTS-2 level) if the location of laboratories were to be aggregated on a higher administrative geographical level for display on the interactive mapping tool (which was done only for Austria, Belgium, Czech Republic, and Poland). All data were anonymous and collected in accordance with the European Parliament and Council decision for the epidemiological surveillance and control of communicable disease in the European community [18],[19]. Ethical approval and informed consent were thus not required.

### Data Analysis, Geographical Illustration, and Cluster Identification

All data were inspected for inconsistencies and analysed on a country-by-country basis and returned to SRLs for feedback, clarification of inconsistencies and final approval in February 2008. After final approval, data were analysed using Stata version 10 and SAS version 9.1 (SAS Institute Inc.) using chi-square test for proportions and Student's *t*-test for continuous variables. The index of diversity is an unbiased measure of the probability of drawing two different *spa* types given the distribution of *spa* types in the sample. The 95% confidence intervals (CIs) were calculated as described previously [20]. Postal address and location of all sampling laboratories were converted into decimal cartesian coordinates using the geocoding facility at www.spatialepidemiology.net [21]. Pairwise distances of laboratories that isolated MSSA and MRSA with identical *spa* types were calculated and distance matrices were summarised and compared by nonparametric tests. The Web application SRL-Maps (http://www.spatialepidemiology.net/srl-maps) was developed to interrogate the data on the basis of mapping of laboratory locations and on centroids of administrative regions (when laboratory results were aggregated at the level of administrative region).

A spatial scan statistic was used to identify the geographic distribution of specific *spa* types at two levels: (i) country-specific frequencies that take into account all *spa* types within national boundaries and (ii) regional clusters of varying size independent of national boundaries. To identify *spa* types with higher than expected occurrence in any of the participating countries, the observed number of all *spa* types isolated within each country was compared with the number expected under the assumption of a European-wide random distribution. For the identification of regional clusters, circular windows around all laboratory locations were projected. For each location, the radius of the window was varied from 0 to 1,000 km. In this way, a finite number of distinct windows was created. For each window, the observed number of isolates with a specific *spa* type was compared with the expected number under the assumption of a random distribution. 10,000 random distributions were obtained by varying the composition of local *spa* types at all laboratory locations consistent with cumulative *spa*-specific frequencies using Monte Carlo Simulations. The test statistic was calculated by log-likelihood ratio test, whereby countries or windows where the observed *spa* type frequencies differed from those obtained by simulation were considered to contain significant clusters with an alpha error of *p*<0.0001. The scan-statistic was executed with SaTScan software using SAS macros [22],[23].

## Results

### Summary Statistics

**Figure 1:**
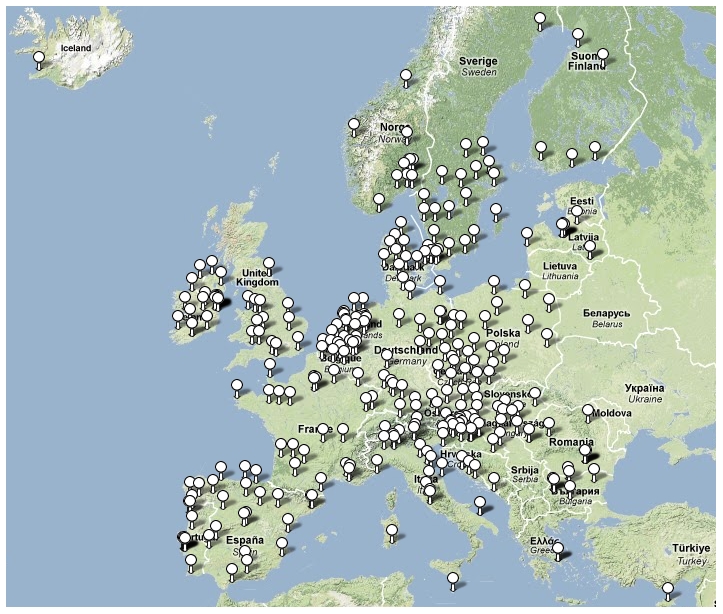
Locations of participating laboratories.

**Figure 2:**
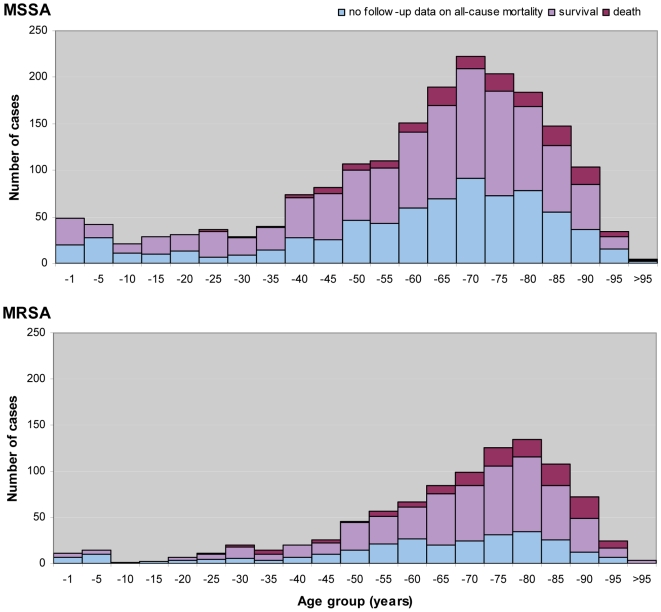
Age distribution and all-cause mortality of patients 14 d after diagnosis of invasive *S. aureus* infections in Europe. Age divided in bands of 5 y, except for infants under 1 y (−1).

26 SRLs from 26 countries satisfactorily fulfilled the proficiency criteria for *spa* sequence typing and contributed data for final analysis. Between September 2006 and February 2007, 357 laboratories serving 450 hospitals (Figure 1) collected 2,890 successive MSSA and MRSA isolates from patients with invasive *S. aureus* infection (2,603 from blood cultures, 90.1%; 17 from cerebrospinal fluid, 0.6%; and 270 from puncture fluids of other normally sterile anatomical sites, 9.3%). Final inspection of data revealed missing information for gender (28 isolates, 1%), age (54 isolates, 1.9%), sampling dates (74 isolates, 2.6%), epidemiological context (community onset or hospital acquisition, 568 isolates 19.7%), all-cause mortality 14 d after *S. aureus* isolation (1052 isolates, 36.4%), and *spa* type (40 isolates, 1.4%). Table 1 gives a summary overview of the number of participating laboratories, isolates, and *spa* types submitted by country. Many laboratories were unable to collect five invasive MRSA isolates within the sampling period because of a low MRSA incidence in the hospitals they serve. Therefore, the combined collection consisted of two-thirds MSSA (1,923; 66.5%) and one-third MRSA (967; 33.5%, Table 2). Patients with invasive MRSA infections were older (Figure 2) with a median age of 69 y compared to a median age of 63 y in MSSA patients (*p*<0.001). Moreover, MRSA patients had higher all-cause mortality (20.8%) 14 d after the first isolation of *S. aureus* than MSSA patients (13.2%, *p*<0.0001). More males (1,765/2,862; 61.7%) than females had invasive *S. aureus* infections. The proportion of MRSA compared to MSSA did not differ between the sexes (*p* = 0.2). Of the 231 MRSA that were reported as community-onset (CO-MRSA), 226 (97.8%) were tested for the presence of PVL-associated genes *luk*-PV and ten (4.4%) were positive. Of the 585 hospital-acquired MRSA (HA-MRSA), 551 (94.2%) were tested for PVL and six (1.1%) were positive. The difference was significant (*p* = 0.009).

**Table 1:** Summary overview of participating laboratories, hospitals, number of invasive isolates of MSSA and MRSA, and *spa* types by country.

Country	*n* Labs	*n* Hospitals	*n* Isolates	MSSA	MRSA^a^	*n spa* Types MSSA	*n spa* Types MRSA	*n* Not Typeable	Percent Not Typed
Austria	18	48	174	120	54	70	19	1	0.6
Belgium	22	22	195	107	88	65	25	1	0.5
Bulgaria	8	8	54	29	25	23	11	0	0.0
Croatia	11	11	88	50	38	27	13	6	6.8
Cyprus	1	1	16	9	7	8	5	0	0.0
Czech Republic	20	20	145	94	51	64	9	0	0.0
Denmark	14	30	112	108	4	72	2	0	0.0
Finland	5	5	22	15	7	14	7	0	0.0
France	23	23	225	114	111	75	27	0	0.0
Germany	27	27	180	98	82	56	20	1	0.6
Greece	3	3	35	20	15	12	6	6	17.1
Hungary	10	13	110	66	44	35	9	2	1.8
Iceland	1	1	5	5	0	5	0	0	0.0
Ireland	22	22	169	85	84	55	26	0	0.0
Italy	19	19	147	80	67	53	15	0	0.0
Latvia	11	12	43	38	5	20	1	0	0.0
Malta	1	1	15	3	12	2	5	3	20.0
Netherlands	18	21	204	195	9	98	9	6	2.9
Norway	11	20	55	55	0	38	0	1	1.8
Poland	23	23	179	132	47	42	14	0	0.0
Portugal	12	12	88	48	40	36	13	0	0.0
Romania	10	10	36	25	11	18	3	0	0.0
Slovenia	11	12	58	48	10	29	3	2	3.4
Spain	21	21	204	113	91	58	19	1	0.9
Sweden	20	47	200	195	5	90	5	3	1.5
UK	15	18	131	71	60	51	18	7	5.3
Total	357	450	2,890	1,923	967	565	155	40	1.4

a The number of MRSA isolates does not reflect a prevalence or occurrence in particular countries as the protocol requested submission of the first five MSSA and MRSA isolates.

**Table 2:** Summary statistics of *S. aureus* isolated in 26 European countries.

Statistics	*n* a	MSSA	MRSA	Total/Overall	*p*-Value*
Frequency (%)	2,890	1,923 (66.5)	967 (33.5)	2,890 (100%)	—
Median age (IQR)	2,836	63 (46–75)	69 (55–78)	66 (49–76)	<0.0001
Male gender (%)	2,862	1,159 (60.8)	606 (63.3)	1,765 (61.7)	0.2
All-cause mortality after 14 d (%)	1,838	153 (13.2)	141(20.8)	294 (16.0)	<0.0001
Hospital acquisition (%)	2,322	777 (51.6)	585 (71.7)	1,362 (58.7)	<0.0001
*N spa* types	2,850	565	155	660^b^	—
*N* not typeable	2,850	27 (1.4)	13 (1.3)	40 (1.4)	0.9
Index of diversity (95% CI)	2,850	0.985 (0.983–0.987)	0.940 (0.933–0.947)	0.983 (0.982–0.984)	<0.05^c^
Mean distance in kilometres between laboratories that isolated identical *spa* types (95% CI)	1,614^d^	1,046.2 (1109.5–983.0)	786.8 (975.7–597.9)	—	0.03^d^

*: *p*-Value for the comparison of MSSA versus MRSA.

a Number of isolates for which data were available.

b Total number of *spa* types includes 60 *spa* types that contain both MSSA and MRSA.

c Deduced from non-overlapping 95% confidence intervals.

d Includes only MRSA and MSSA with more than ten isolates per *spa* type.

IQR, interquartile range.

### *spa* Typing

**Figure 3:**
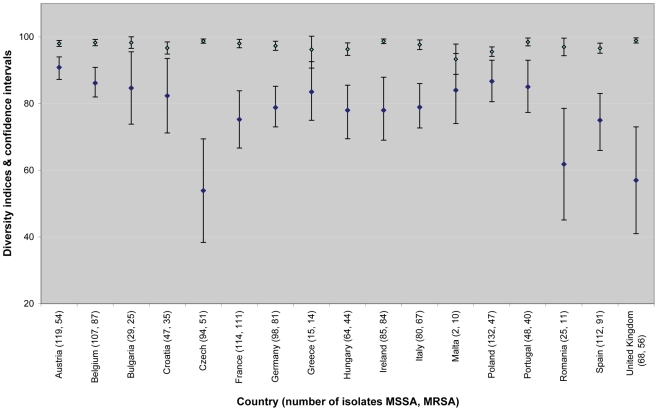
Estimates of country-specific genetic diversity expressed as Simpson's index of diversity of *spa* types (as a percentage) for MSSA (light blue diamonds) and MRSA (dark blue diamonds) and 95% CIs (bars). Only countries for which *spa* type information for more then ten MRSA isolates were available were included in this figure.

A total of 660 *spa* types were reported (Table 2). Among all *spa* types, 565 and 155 were assigned to MSSA and MRSA, respectively, of which, 505 were exclusively identified as MSSA and 95 for MRSA alone. 60 *spa* types contained both MSSA and MRSA. 27 of the MSSA (1.4%) and 13 of the MRSA (1.3%) isolates were nontypeable. Table 3 shows the rank order of the most frequent *spa* types isolated during the survey and Table 4 the three most frequent *spa* types by country. MRSA was less diverse than MSSA. Only five *spa* types accounted for almost half (48.1%) of all MRSA isolates, whereas the same proportion of MSSA isolates comprised the 26 most frequent MSSA *spa* types. Estimates of the genetic diversity differed significantly with an index of diversity for MSSA of 0.985 (95% CI 0.983–0.987) and 0.940 (95% CI 0.933–0.947) for MRSA. While MSSA diversity ranged between 0.934 in Latvia and 1.0 in Iceland (unpublished data), MRSA diversity between countries was more heterogeneous ranging from 0.62 in Romania to 0.91 in Austria (Figure 3), indicating the presence of few dominant MRSA *spa* types in several countries. Accordingly, MRSA showed a higher degree of geographic clustering as the average distance between laboratories that isolated the same *spa* type was significantly smaller than for MSSA (Table 2). No correlation between genetic diversity of MRSA and overall proportion of MRSA among *S. aureus* blood stream infections at country level as reported to the EARSS database for 2007 was found (*r* = −0.09, *p* = 0.75) [24], indicating that single successful *spa* types cannot explain the variance in the proportion of MRSA causing *S. aureus* blood stream infections observed in Europe.

**Table 3:** 20 most frequent *spa* types and their STs among MSSA and MRSA isolated in 26 European countries.

Rank	MSSA		Frequency	Percent	Cumulative Percent	Rank	MRSA		Frequency	Percent	Cumulative Percent
	*spa* Type	MLST					*spa* Type	MLST			
**1**	**t002**	ST-5^a^, S-231^a^	93	4.8	4.8	**1**	**t032**	ST-22^a^	140	14.5	14.4
**2**	**t084**	ST-15^a^ (ST-18)	89	4.6	9.5	**2**	**t008**	ST-8^a^ (ST-247, ST-250, ST-254)	120	12.4	26.8
**3**	**t015**	ST-45^a^	84	4.4	13.8	**3**	**t041**	ST-111^a^, ST-228^a^	72	7.4	34.2
**4**	**t091**	ST-7^a^	82	4.3	18.1	**4**	**t003**	(ST-5) ST-225^a^	71	7.3	41.6
**5**	**t012**	ST-30^a^	77	4.0	22.1	**5**	**t002**	ST-5^a^, ST-231^a^	62	6.4	48.1
**6**	**t127**	ST-1^a^	57	3.0	25.1	**6**	**t067**	ST-5^a^, ST-125^a^	50	5.2	53.3
**7**	**t008**	ST-8^a^ (ST-247, ST-250, ST-254)	55	2.9	27.9	**7**	**t001**	(ST-5, ST-222) ST-228^a^	30	3.1	56.4
**8**	**t021**	ST-30^a^ (ST-33, ST-55)	49	2.5	30.5	**8**	**t037**	ST-239^a^ (ST-240), ST-241^a^	27	2.8	59.2
**9**	**t005**	ST-22^a^ (ST-23, ST-60)	42	2.2	32.7	**9**	**t030**	ST-239^a^ (ST-246)	20	2.1	61.2
**10**	**t026**	(ST-45, ST-47)	27	1.4	34.1	**10**	**t024**	ST-8^a^	14	1.4	62.7
**11**	**t065**	(ST-45, ST-46)	26	1.4	35.4	**11**	**t190**	ST-8^a^	14	1.4	64.1
**12**	**t160**	(ST-12, ST-13)	26	1.4	36.8	**12**	**t515**	ST-22^a^	12	1.3	65.5
**13**	**t056**	(ST-101)	25	1.3	38.1	**13**	**t038**	ST-45^a^	12	1.2	66.7
**14**	**t050**	ST-45^a^	21	1.1	39.2	**14**	**t022**	ST-22^a^	11	1.1	67.8
**15**	**t078**	(ST-26)	21	1.1	40.2	**15**	**t740**	ST-45^a^	11	1.1	69.0
**16**	**t164**	(ST-20)	19	1.0	41.2	**16**	**t012**	ST-30^a^	9	0.9	69.9
**17**	**t346**	(ST-15, ST-620)	18	0.9	42.2	**17**	**t015**	ST-45^a^	9	0.9	70.8
**18**	**t024**	ST-8^a^	17	0.9	43.1	**18**	**t044**	ST-80^a^	9	0.9	71.8
**19**	**t230**	ST-45^a^	17	0.9	43.9	**19**	**t045**	ST-5^a^ (ST-225)	8	0.8	72.6
**20**	**t166**	(ST-34)	16	0.8	44.8	**20**	**t127**	ST-1^a^	8	0.8	73.4
**—**	**Other**	—	1,062	55.2	100.0	—	**Other**	—	258	26.6	100.0
**Total**	**—**	—	1,923	100	—	**—**	**—**	—	967	100	—

STs in parentheses are those associated with the spa type in the SeqNet.org Spa typing data base.

a MLST as determined by SRLs.

**Table 4:** First, second, and third most frequent MSSA and MRSA *spa* types per country and their relative proportions.

Country	MSSA *spa* Type				MRSA *spa* Type			
	*n*	1st (%)	2nd (%)	3rd (%)	*n*	1st (%)	2nd (%)	3rd (%)
**Austria**	120	t091 (8.3)	t002 (6.7)	t012 (5.0)	54	t190 (18.5)	t041 (16.7)	t001 (14.8)
**Belgium**	107	t002 (9.4)	t209 (4.7)	t012, t091, t740 (3.7)	88	t008 (29.6)	t002, t038 (13.7)	t740 (12.5)
**Bulgaria**	29	t056 (10.3)	t078, t148, t156, t1346 (6.9)	a	25	t030 (36.0)	t037 (16.0)	t010 (12.0)
**Croatia**	50	t050 (10.0)	t005, t015, t1361 (8.0)	t164 (6.4)	38	t041 (36.8)	t091 (10.5)	t026, t1003 (8.3)
**Cyprus**	9	t002 (22.2)	a	—	7	t012, t30 (28.6)	a	—
**Czech Republic**	94	t015, t130 (5.3)	t024, t122, t1081 (4.3)	t056, t156, t491, t1231 (3.2)	51	t003 (66.7)	t032 (13.7)	t002 (7.8)
**Denmark**	108	t230 (7.4)	t002, t127 (4.6)	t065, t084 (3.7)	4	t024, t037 (50.0)	—	—
**Finland**	15	t026 (13.3)	a	—	7	b	—	—
**France**	114	t002 (11.4)	t008 (6.1)	t012 (4.4)	111	t008 (48.6)	t777 (7.2)	t024 (5.4)
**Germany**	98	t008 (11.2)	t084 (7.1)	t015, t091 (6.1)	82	t032 (35.4)	t003 (28.1)	t001 (8.6)
**Greece**	20	t267 (15.0)	t012 (10.0)	a	15	t002, t044 (26.7)	t037 (20.0)	a
**Hungary**	66	t091, t216 (10.6)	t012, t084 (7.60)	t002, t015, t2115 (4.7)	44	t032 (38.6)	t041 (25.0)	t062 (13.6)
**Iceland**	5	b	—	—	0	—	—	—
**Ireland**	85	t021 (7.1)	t012 (4.7)	t078, t127, t166, t382, t548 (3.6)	84	t032 (45.2)	t515 (9.5)	t022 (4.8)
**Italy**	80	t091 (10.04)	t084 (8.8)	t012 (7.5)	67	t041 (34.3)	t008 (28.4)	t001 (13.4)
**Latvia**	38	t435 (21.1)	t015 (13.2)	t313, t698 (7.9)	5	b	—	—
**Malta**	3	b	—	—	12	t001, t032 (30.0)	t012 (20.0)	t002, t022 (10.0)
**Netherlands**	195	t091 (7.7)	t127 (6.2)	t002, t012, t084 (4.2)	9	b	—	—
**Norway**	55	t065 (9.1)	t084 (9.1)	t002, t015, t095 (3.7)	0	—	—	—
**Poland**	132	t127 (12.9)	t084 (9.9)	t015 (7.6)	47	t037 (29.81)	t003, t015 (14.9)	t002, t041, t1574 (6.4)
**Portugal**	48	t008 (8.3)	t002, t645 (6.3)	t021, t127, t148, t148, t179, t189 (4.2)	40	t032 (32.5)	t002 (20.0)	t535, t747, t2357 (7.5)
**Romania**	25	t021, t284 (12.0)	t005, t008, t450 (8.0)	a	11	t030 (54.6)	t127 (36.4)	t015 (9.1)
**Slovenia**	48	t091 (20.8)	t015 (10.4)	t005 (8.5)	10	t041 (70.0)	a	—
**Spain**	113	t002 (12.4)	t012, t067 (8.0)	t015 (4.5)	91	t067 (47.3)	t002 (15.4)	t008 (7.7)
**Sweden**	195	t015 (9.2)	t084 (8.2)	t012 (5.7)	5	b	—	—
**UK**	71	t012, t127 (5.6)	t021 (4.2)	nine different types	60	t032 (61.7)	t788, t1516 (3.3)	a

a All remaining spa types equally distributed.

b No ranking, all spa types equally distributed.

### Clustering of *spa* Types at Country and Regional Level

**Figure 4:**
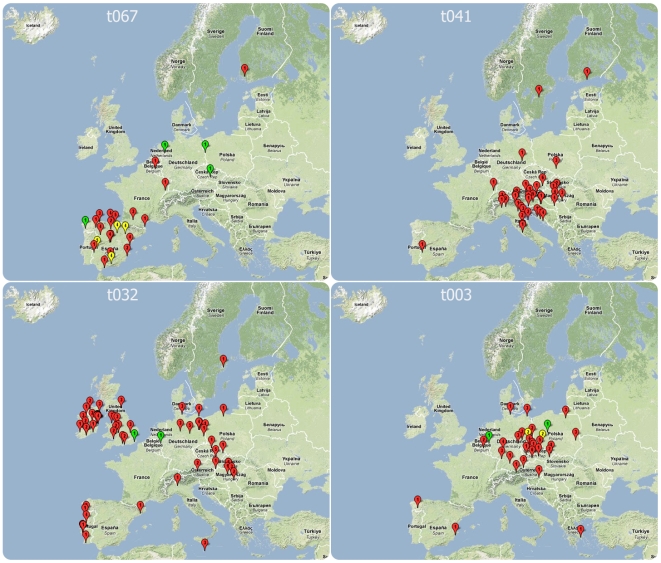
Location of laboratories isolating *S. aureus* of *spa* types t067, t041, t032, and t003, which are the four most significant regional clusters on SRL-Maps. The numbers within each placemark represent the number of isolates and the colours represent resistance phenotypes: red, MRSA; green, MSSA; yellow, a mixture of MRSA and MSSA.

In 17 countries, 22 *spa* types were found with frequencies that were unexpected when applying the hypothesis of a random distribution, indicative of local epidemics (Table 5). Most (86.9%) of these were MRSA. In ten countries two *spa* types coexisted with unexpected frequencies. In four of them, these two *spa* types showed a close genetic relationship and belonged to the same *spa* complex whereby a single genetic event could account for the sequence divergence between the types. There was also a frequent regional coincidence with neighbouring countries sharing identical epidemic *spa* types. The Czech Republic and Germany shared *spa* type t003 (t003/ST225/SCC*mec*II), Bulgaria and Romania shared t030 (t030/ST239/SCC*mec*III), the UK and Ireland t032 (t032/ST22/SCC*mec*IV), Italy and Croatia shared t041 (t041/ST228/SCC*mec*I), and strain t067 (t067/ST5125/SCC*mec*IV) whose dominance in Spain was first identified through this initiative [25], was also found in southern France. The notion of regional spread was supported by the cluster statistic that projected windows beyond national boundaries for this dataset (Table 6). The degree of unexpectedness, which is an indication of the significance of each cluster, is expressed by the log likelihood ratio (LLR). The majority of regional clusters extended beyond national boundaries and 74% of all isolates that occurred in these clusters were MRSA. The most significant cluster was identified in Spain and consisted of *spa* type t067 (t067/ST5&125/SCC*mec*IV). A northern Balkan/Adriatic cluster consisting of *spa* type t041 (t041/ST228/SCC*mec*I) was found in Austria, Hungary, Slovenia, Croatia, and northern and central Italy. In Britain and Ireland, t032 (t032/ST22/SCC*mec*IV), known as epidemic MRSA 15 (EMRSA-15), was the dominant type and represented the third most significant cluster. An additional cluster of *spa* type t032, albeit less significant and much smaller, was located in the Brandenburg area of Germany. Central Germany, the Czech Republic, and western Poland were included in a large regional cluster of *spa* type t003 (t003/ST225/SCC*mec*II), which was geographically centred in Saxony and had a radius of approximately 400 km corresponding to the German hospitals participating in the study. Figure 4 provides a geographical illustration of these clusters. The largest cluster in size as well as in numbers (radius 930 km, 119 isolates) consisted of *spa* type t008 and was centred in southern France. This cluster consisted of ST8 and contained different subclones as it included both MSSA and MRSA, and MRSA isolates exhibited two different *SCCmec* elements (*SCCmec*I and IV). A smaller cluster, ranking in sixth position in terms of significance, was located in Flanders on the Belgian-Dutch border and consisted of *spa* type t740 (t740/ST45/SCC*mec*IV). Interestingly, regional *spa* clusters with overlapping geographical range were frequently made up of *spa* types that belonged to the same *spa* complex, a clear indication that local spread is accompanied by local evolution of the rapidly evolving *spa* locus. Clusters with the smallest size (0 km) included those submitted by single laboratories most likely reflecting single hospital outbreaks. Three regional clusters consisted of MSSA alone. They were located in Latvia (t435/ST425), Poland (t127/ST1), and Denmark (t230/ST45), indicating that regional spread of *S. aureus* is not limited to MRSA alone.

**Table 5:** Unexpectedly frequent *spa* types at country level assuming a European-wide random distribution.

Country Cluster Number	Country	*spa* Type	*spa* Complex^a^	ST^b^	*n* Labs Reporting Clustered *spa* Type	*n* Labs Participating in Survey	Percent Labs Reporting Clustered *spa* Type	*n* Isolates Observed	*n* Isolates Expected^c^	*n* MRSA among Observed Isolates	Percent MRSA among Observed Isolates
**1**	**Austria**	t190	190	8	7	18	38.9	11	0.9	10	91
**2**	**Belgium**	t740	740	45	6	22	27.3	15	1.0	11	73
**3**	**Belgium**	t038	740	45	8	22	36.4	12	0.8	12	100
**4**	**Bulgaria**	t030	12	239	4	8	50.0	10	0.4	9	90
**5**	**Croatia**	t041	1	228	7	11	63.6	14	2.2	14	100
**6**	**Czech Republic**	t003	45	225	16	20	80.0	34	3.8	34	100
**7**	**Czech Republic**	t130	130	—	3	20	15.0	5	0.4	5	100
**8**	**Denmark**	t230	728	45	7	14	50.0	8	0.7	0	0
**9**	**France**	t008	8	8	22	23	95.7	61	13.6	54	89
**10**	**France**	t777	777	5	6	23	26.1	9	0.7	8	89
**11**	**Germany**	t003	45	225	10	27	37.0	24	4.7	23	96
**12**	**Germany**	t032	32	22	9	27	33.3	29	9.5	29	100
**13**	**Greece**	t044	44	80	3	3	100.0	5	0.1	4	80
**14**	**Hungary**	t062	Singleton	5	2	10	20.0	6	0.3	6	100
**15**	**Hungary**	t216	Singleton	59	7	10	70.0	7	0.5	7	100
**16**	**Ireland**	t032	32	22	18	22	81.8	38	8.9	38	100
**17**	**Ireland**	t515	32	22	7	22	31.8	8	0.7	8	100
**18**	**Italy**	t041	1	228	13	19	68.4	23	3.7	23	100
**19**	**Italy**	t001	1	228	8	19	42.1	9	1.5	9	100
**20**	**Latvia**	t435	435	427	4	11	36.4	8	0.2	8	100
**21**	**Latvia**	t425	425	368	3	11	27.3	5	0.1	5	100
**22**	**Poland**	t037	12	239	11	23	47.8	21	2.2	14	67
**23**	**Poland**	t127	127	1	6	23	26.1	17	4.0	17	100
**24**	**Romania**	t030	12	239	3	10	30.0	6	0.3	6	100
**25**	**Spain**	t067	2	5 & 125	18	21	85.7	52	4.4	43	81
**26**	**Spain**	t002	2	5	15	21	71.4	28	10.9	14	50
**27**	**United Kingdom**	t032	32	22	12	15	80.0	39	6.9	27	96
**Total**	**—**	—	—	—	235	—	—	504	83.4	438	87

a *spa* complexes group *spa* types that differ by only a single evolutionary event (single indel or nucleotide polymorphism) into the same complex.

b As determined by SRLs.

c Average number of isolates with this *spa* type expected in country after 10,000 simulations on the basis of European-wide cumulative frequency.

**Table 6:** Regional clusters of *spa* types.

Regional Cluster Number	*spa* Type	*spa* Complex^a^	ST^b^	Window Centre	Window Radius (km)	Countries reporting Clustered *spa* Type within Window	*n* Isolates Observed	*n* Isolates Expected^c^	Log Likelihood Ratio	*n* MRSA among Observed Isolates	Percent MRSA among Observed Isolates
**1**	**t067**	2	5 & 125	Alicante, Spain	716	ES, FR	55	4.7	126.9	46	84
**2**	**t041**	1	228	Split, Croatia	522	AT, HR, HU, SI, IT	59	10.6	84.76	59	100
**3**	**t032**	32	22	Belfast, Northern Ireland (UK)	596	IE, UK	77	15.9	84.74	75	97
**4**	**t003**	45	225	Leipzig, Germany	386	CZ, DE, PL	58	10.0	82.26	54	93
**5**	**t008**	8	8	Perpignan, France	931	AT, BE, DE, ES, FR, HR, PT, SI	119	45.5	72.96	105	88
**6**	**t740**	740	45	Goes, Netherlands	81	BE, NL	15	0.6	50.36	11	73
**7**	**t030**	12	239	Pleven, Bulgaria	331	BG, RO	16	0.7	45.24	15	94
**8**	**t037**	12	239	Plock, Poland	330	PL	21	2.2	36.02	18	86
**9**	**t038**	740	45	Wilrijk, Belgium	92	BE	12	0.8	32.71	12	100
**10**	**t190**	190	8	St Pölten, Austria	56	AT	10	0.3	29.63	9	90
**11**	**t001**	1	228	Sibenik, Croatia	885	AT, DE, HU, IT, MT	29	10.2	27.55	29	100
**12**	**t435**	435	427	Daugavpils, Latvia	189	LV	8	0.2	25.82	0	0
**13**	**t425**	239	368	Riga, Latvia	0	LV	5	0.1	22.76	5	100
**14**	**t127**	127	1	Inowroclaw, Poland	131	PL	12	0.9	22.65	0	0
**15**	**t032**	32	22	Berlin, Germany	128	DE	19	3.2	22.2	19	100
**16**	**t015**	15	45	Lomza, Poland	581	CZ, LV, PL	35	11	20.72	7	20
**17**	**t091**	91	7	Ried, Austria	771	AT, BE, CZ, DE, FR, HR, HU, IT, NL, PL, SI	71	42.8	20.68	4	6
**18**	**t777**	777	5	Laval, France	277	FR	7	0.3	20.61	7	100
**19**	**t081**	78	25	Maków Mazowiecki, Poland	0	PL	4	0.0	19.37	3	75
**20**	**t515**	32	22	Mullingar, Ireland	196	IE, Northern Ireland (UK)	9	0.7	18.94	9	100
**21**	**t002**	2	5, 231	Coimbra, Portugal	484	ES, PT	33	10.6	18.37	20	61
**22**	**t230**	728	45	Hilleroed, Denmark	235	DE, DK, SE	11	1.4	17.19	0	0
**23**	**t044**	44	80	Nicosia, Cyprus	916	GR, CY	6	0.2	17.09	6	100
**24**	**t2054**	8	8	St Mande, France	226	BE, FR4	5	0.2	16.64	3	60
**25**	**t062**	singleton	5	Szolnok, Hungary	145	HU	6	0.3	15.24	6	100
**Total**	**—**	—	—	—	368 (mean)	339	702	173.1	—	522	74

Analyses were performed for *spa* types with five or more isolates during the study period.

a *spa* complexes group *spa* types that differ by only a single evolutionary event (single indel or nucleotide polymorphism) into the same complex.

b As determined by SRLs.

c Average number of isolates with this spa type expected in window after 10,000 simulations on the basis of European-wide cumulative frequency.

AT, Austria; BE, Belgium; BG, Bulgaria; CY, Cyprus; CZ, Czech Republic; DE, Germany; DK, Denmark; ES, Spain; FI, Finland; FR, France; GR, Greece; HR, Croatia; HU, Hungary; IE, Ireland; IT, Italy; LV, Latvia; MT, Malta; NL, Netherlands; PL, Poland; PT, Portugal; RO, Romania; SE, Sweden, SI, Slovenia; UK, United Kingdom.

### SRL-Maps

**Figure 5:**
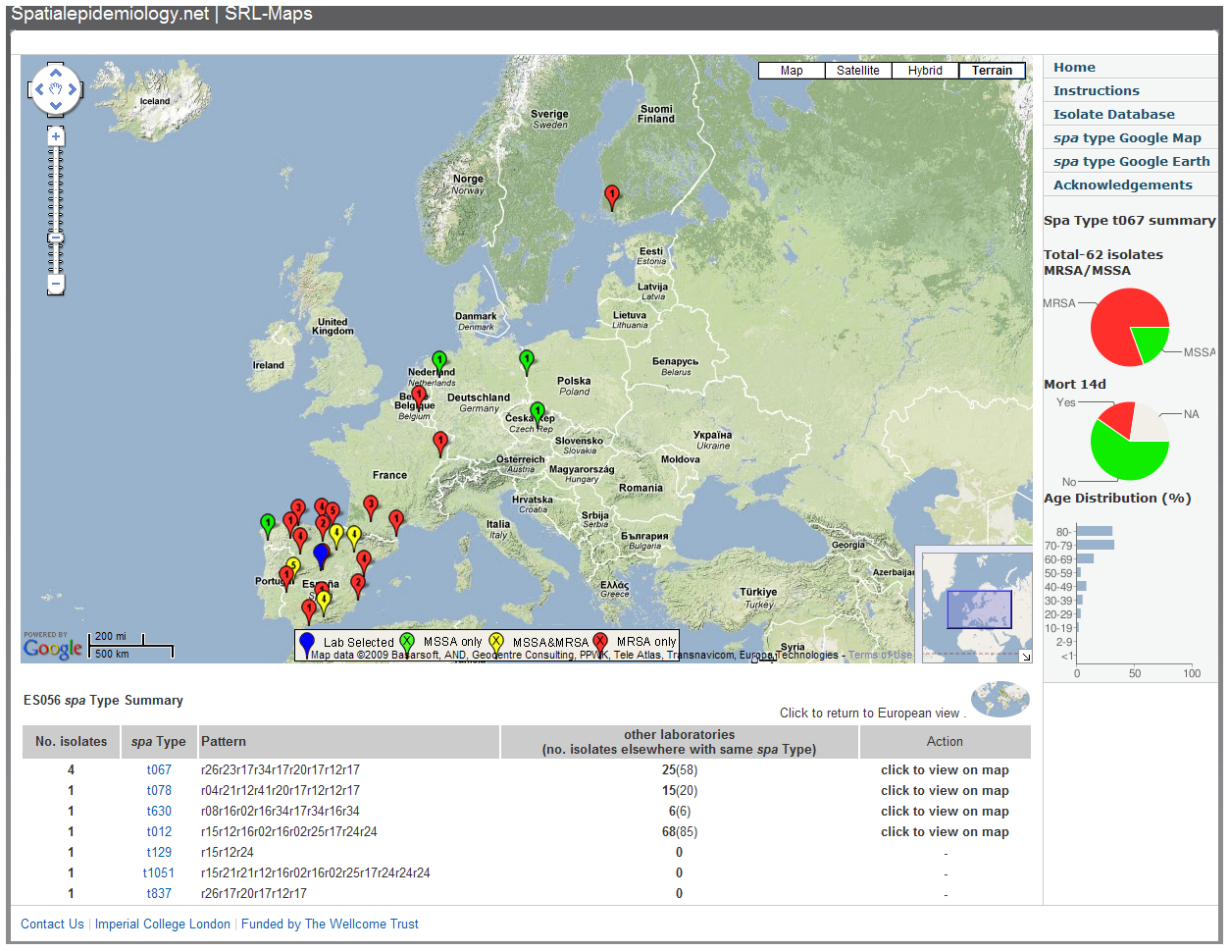
Location of laboratories isolating *S. aureus* spa type t067 (as shown in Figure 4) viewed using SRL-Maps. Isolates from LAB ES056 in Madrid, Spain, and the distribution of all other t067 isolates are shown (*n* = 62). Each placemark indicates whether isolates are MSSA (green), MRSA (red), or a mixture (yellow) and the selected laboratory is blue. The numbers of isolates are indicated inside the placemark. The pie charts on the right show the proportion of MRSA/MSSA and all-cause mortality after 14 d, and the bar chart displays patient age distribution.

The Web application SRL-Maps (http://www.spatialepidemiology.net/srl-maps) provides a community tool for the interrogation of the geographic distribution of different *spa* types. All laboratory and regional locations across Europe are represented as placemarks on a Google map. Clicking on a placemark displays, below the map, all *spa* types identified at that location (and their frequency) along with the number of isolates (and number of geographic locations) found elsewhere (if any) for each of these *spa* types (Figure 5). The European distribution of any *spa* type can then be displayed and placemarks on the map are colour-coded on the basis of whether the isolates at each location are all MSSA (green), all MRSA (red), or are a mix of MSSA and MRSA (yellow), with the number of isolates inside the placemark. Graphical charts are displayed showing *spa* type-specific proportion of MRSA/MSSA, all-cause mortality at 14 d, and the age distribution among cases (Figure 5). This method allows the identification and mapping of strains with particular public health importance and further exploration by the scientific community is encouraged.

### Observations on Specific *spa* Types

For both MSSA and MRSA isolates, there was no association between specific *spa* types and all-cause mortality after 14 d, indicating that no *spa* type was associated with hyper-virulence. Of the ten CO-MRSA isolates that were found to be PVL-positive, three were assigned to *spa* type t044 (t044/ST80/SCC*mec*IV), the so-called European community-acquired (CA)–MRSA, and another three had *spa* type t008 (t008/ST8/SCC*mec*IV) and are indistinguishable from USA300 CA-MRSA. Of the four other PVL-positive CO-MRSA, two belonged to t622 (*spa* complex 8/ST8/SCC*mec*IV), one to t529 (ST1043/SCC*mec*V), and one to t437 (not further characterised). MRSA belonging to ST398 have recently emerged in several European countries and are regarded as being livestock-associated (LA-MRSA). Of all *spa* types typically associated with this clone, *spa* types t011, t034, t571, t1255, and t2383 were identified on 12 occasions (1.3%) in eight countries during this survey. None of these isolates, however, displayed an MRSA phenotype or harboured the *mecA* gene.

## Discussion

Predominant *spa* types showed a wide geographic distribution. The average distance between the locations from which the same *spa* types were sampled was smaller for MRSA isolates than MSSA isolates, suggesting a higher degree of geographical clustering of MRSA. Moreover, genetic diversity was much lower for invasive MRSA than MSSA and differed considerably between countries. Spatial-scan statistics corroborated a fundamental difference between MRSA and MSSA with respect to regional dissemination. The majority of isolates that formed regional clusters were MRSA, and 13 of the 15 major MRSA *spa* types (defined as more than ten isolates in the database) occurred in geographical clusters. They were typically hospital acquired and no more than three clusters overlapped in the same region. Conversely, of the 27 major MSSA *spa* types, only five showed significant geographical clustering and only three consisted of MSSA alone. Thus, invasive MRSA clones in Europe display a typical epidemic behaviour and have a predominantly regional distribution. To unravel the dynamics of spread of these epidemic MRSA requires the present type of survey to be repeated every few years.

The emergence of MRSA occurs by the acquisition of the methicillin resistance determinant (SSC*mec*) by MSSA strains. MRSA strains typically emerge from the most prevalent MSSA strains and it is a rare event, although new findings suggest that it is more frequent than originally suggested [26],[27]. There are thus fewer MRSA clones compared to MSSA clones and they are very young on evolutionary time scales (less than 50 y old) and have had little time to diversify since they arose, whereas MSSA are much older and thus more diverse. MRSA clones also expand because of the selective forces exerted by heavy antibiotic use in hospitals and conditions that favour transmission within and between hospitals, which constrains their diversity. In contrast, MSSA will be subject to much weaker selection leading to neutral genetic drift that maintains their diversity. Geographic spread of MRSA clones will be facilitated by repeated hospital admissions and referrals of MRSA carriers [28] who typically belong to an older and thus less mobile segment of the population. The broader distribution of MSSA clones may reflect dissemination by hosts with different travel patterns than MRSA carriers as well as the longer time that MSSA clones have had to spread.

The present survey set several precedents in the field of molecular epidemiology of *S. aureus*. First, it brought together reference laboratories from 26 European countries adopting a standardised quality-controlled DNA sequence-based typing method; second, it provided a contemporaneous and comprehensive population snapshot of *S. aureus* isolates from invasive disease using an agreed sampling frame; third, it utilised modern spatial scan statistics to identify geographical clustering; fourth, it provided the first public domain Web-based interactive mapping tool for future public health research; and finally, it consolidated a collaborative framework for the continuation of this important European surveillance initiative.

All Member States of the European Union except Estonia, Lithuania, Luxemburg, and Slovakia participated and variously achieved a country-wide enrolment of diagnostic laboratories. In the run-up to this study, a successful effort was undertaken to agree on a single molecular typing approach to scale up the typing capacity, and improve quality assessment, by introducing proficiency testing for SRLs that intended to participate in advanced *S. aureus* surveillance. This effort provided the basis for the execution of a mutually agreed protocol using a standardized sampling frame and a quality-controlled genetic typing approach [12],[15], based on the sequencing of the variable region of the *S. aureus spa* gene (*spa* typing) [11]. Multilocus sequence typing (MLST) was also carried out on many strains allowing most of the prevalent *spa* types to be related to MLST sequence types. However, given the scope and ambition of this investigation, it is not surprising that the study still suffered from several operational shortcomings.

In order to keep the amount of work manageable for the participating SRLs, it was decided to include about 20 laboratories that were demographically and geographically representative of each of the participating countries. This number is arbitrary and cannot equally represent small and large countries alike. Thus the precision of spatial scan statistics is reduced in areas where the density of laboratories is low. Laboratory enrolment based on population size would provide a more appropriate sampling strategy but would also impose a proportional and frequently unacceptable amount of work on SRLs in large countries if the sample size from small countries should remain meaningful. Even medium-sized countries such as Bulgaria, Finland, Greece, and Romania were unable to enrol the intended number of laboratories mainly for technical and logistic reasons. Naturally, the number of laboratories and isolates varied between countries and geo-demographic representation could be improved in future investigations. The original intention was to collect equal numbers of successive MSSA and MRSA in all laboratories during the 6-mo sampling interval; this proved to be unrealistic, especially in countries where MRSA levels are low. As a result, Norway and Iceland could not provide any MRSA, whereas Sweden, Denmark, and the Netherlands each provided fewer than ten isolates. Cyprus and Malta had only one participating laboratory but since both provide the microbiological service for the whole of the respective island population (for Cyprus, only the Republic of Cyprus), they were entitled to submit up to 20 isolates. Nevertheless, even taking these potential problems into account, the simultaneous collection of 2,890 isolates from patients with invasive *S. aureus* infection treated in 450 hospitals during a 6-mo study interval is unparalleled and remains unmatched by previous investigations, which have drawn their conclusions from convenience samples of predominantly MRSA collected by laboratories for different clinical or biological reasons. The current collection includes one-third MRSA and thus over-represents the natural population of MRSA causing invasive disease, which was 22% in the European Union in 2007 [24]. All isolates were collected from laboratories and hospitals participating in EARSS for which estimates of the overall catchment population are known. Thus, the present sample of hospitals catered for approximately 22 million people, totalling 4.4% of the citizens living in the European Union.

Despite the above limitations, the sample provides a realistic insight into the epidemiology of *S. aureus* currently causing invasive infection in Europe. The age distribution and all-cause mortality was consistent with the expected range [29],[30]. High frequencies of invasive infections were ascertained in the very young (infants and under 5-y-olds) and older age groups with males clearly more at risk than females. Patients with MRSA were older than patients with MSSA and were 2.4 times more likely to have their infection attributed to hospital acquisition (*p*<0.0001). Invasive MRSA carried a higher all-cause mortality after 14 d. This finding is most likely confounded by a host of variables that distinguish MRSA patients from MSSA patients in fundamental ways. All-cause mortality was independent of *spa* type, indicating that this study did not identify any single *spa* type that stands out with respect to hyper-virulence or other factors that would increase the risk of fatal outcome after 14 d. While a laboratory-based cross-sectional study is limited in its ability to control for many of the crucial confounders such as comorbidity and disease severity scores and thus may not detect subtle differences in virulence properties between different clonal lineages at the patient level, this sample provides an unbiased estimate of the frequencies of specific *spa* types that have previously been reported to cause outbreaks and have attracted considerable public health attention. Few MRSA isolates carried the PVL-toxin genes and this could be an indication that many CO-MRSA were originally hospital-acquired. Only six of the PVL-positive CO-MRSA isolates, which made up 0.5% of the overall sample, had *spa* types consistent with the CA-MRSA clones most frequently reported in Europe (t008/ST8/SCC*mec*IV and t044/ST80/SCC*mec*IV) [31]. These values when compared to the overall numbers of MRSA are small but still warrant attention since PVL-positive CA-MRSA are more commonly associated with skin and soft tissue infections and are rarely found in blood stream infections [31] from which the majority of the sample isolates were drawn. Livestock-associated *spa* types belonging to MLST sequence type ST398 [32] made up 0.4% of the overall sample. However, none were methicillin-resistant indicating a low rate of human systemic infections induced by MRSA variants of this clone despite the increasing interest and concern about such isolates by health authorities.

With the decision to utilize *spa* typing as a common platform to address the geographic abundance of *S. aureus* clones, various potential problems that might affect observations need to be taken into account. First, a single sequence of approximately 280 base-pairs under potential immune selection may be a relatively weak indicator for the genetic background of a genome that is approximately 10,000-times larger, even in a species such as *S. aureus* that is evolving in a predominantly clonal manner. Furthermore, convergent evolution could occur as a result of the high mutability of the repeat region of the *spa* gene used in *spa* typing [26],[33]. Finally, *spa* typing on its own it may not be sufficiently discriminatory to distinguish between MRSA isolates given that only five *spa* types accounted for almost half (48.1%) of all 967 MRSA isolates examined in this survey. The regional clusters found in this study provide a good indication that homoplasy is not a major problem that hinders the recognition of clonal dissemination on a geographic scale. Moreover, when comparing *agr* type, SCC*mec* type, toxin gene, and antibiotic susceptibility profiles within and between different *spa* types, a significant within-*spa*-type consistency and between-*spa*-type discordance supports the notion that in most cases *spa* typing provides a convenient and valid marker for the major clones and clonal lineages [34].

In order to explore the geographic distribution of different *spa* types, and the genetic and phenotypic detail of isolates in the European sample, the reader is encouraged to explore the purpose-built interactive Web application, SRL-Maps, at http://www.spatialepidemiology.net/srl-maps/. This application contains the public domain data made available to the scientific and public health community by all SRLs that participated in the study. It also illustrates the potential of such a communication platform. The underlying database is fully searchable and the map-based application is a template for the future addition of further epidemiological and biological information. We believe that this approach to spatial epidemiology will become a rich resource for future enquiry into the population dynamics of infectious agents and their evolution.

In conclusion we provide evidence that the major MRSA clones in Europe occur predominantly in geographical clusters. This also indicates that MRSA, rather than spreading freely in the community, diffuses through regional health care networks. This important finding suggests that control efforts aimed at interrupting the spread within and between health care institutions may not only be feasible but ultimately successful and should therefore be strongly encouraged. We also showed that an international surveillance network sharing decentralised typing results on a Web-based platform can provide crucial information for clinicians, diagnostic microbiologists, and infection control teams on the dynamics of *S. aureus* spread, and especially the spread of MRSA isolates, to provide early warning of emerging strains, cross-border spread, and importation by travel.

## Supporting Information

Text S1 Affiliations and contact information of the *Staphylococcus aureus* Reference Laboratory Working Group members.
(0.16 MB DOC)

Text S2 Author contributions of the *Staphylococcus aureus* Reference Laboratory Working Group members.
(0.13 MB DOC)

## Abbreviations

CA-MRSA: community-acquired methicillin-resistant *Staphylococcus aureus*
CI: confidence interval
CO-MRSA: community-onset methicillin-resistant *Staphylococcus aureus*
EARSS: European Antimicrobial Resistance Surveillance System
MLST: multilocus sequence typing
MRSA: methicillin-resistant *Staphylococcus aureus*
MSSA: methicillin-sensitive *Staphylococcus aureus*
PVL: Panton-Valentine Leukocidin
SRL: *S. aureus* Reference Laboratories
ST: sequence type
