# Supplementary material for: Geographic Distribution of Staphylococcus aureus Causing Invasive Infections in Europe: A Molecular-Epidemiological Analysis
Source: PLoS Med. 2010 Jan 12;7(1):e1000215. doi: 10.1371/journal.pmed.1000215 (PMC2796391; doi:10.1371/journal.pmed.1000215)
Supplement: Text S1 — Affiliations and contact information of the Staphylococcus aureus Reference Laboratory Working Group members. (0.16 MB DOC) [file pmed.1000215.s001.doc]

**Text S1. The *Staphylococcus aureus* Reference Laboratory Working Group: Affiliations and Contact Information**

| Hajo Grundmann, | (1) National Institute for Public Health and the Environment, Bilthoven, The Netherlands | hajo.grundmann@rivm.nl |
| --- | --- | --- |
|  | (2) Department of Medical Microbiology, University Medical Centre, Groningen, The Netherlands | |
| David M Aanensen, | Department of Infectious Disease Epidemiology, Imperial College, London, UK | d.aanensen@imperial.ac.uk |
| Cees C. van den Wijngaard, | National Institute for Public Health and the Environment, Bilthoven, The Netherlands | Kees.van.den.Wijngaard@rivm.nl |
| Artur J. Sabat, | Department of Medical Microbiology, University Medical Centre | Artur Sabat <A.J.Sabat@med.umcg.nl> |
| Jan Muilwijk, | National Institute for Public Health and the Environment, Bilthoven, The Netherlands | Jan.Muilwijk@rivm.nl |
| Jos Monen, | National Institute for Public Health and the Environment, Bilthoven, The Netherlands | jos.monen@rivm.nl |
| Adriana Tami, | Department of Medical Microbiology, University Medical Centre, Groningen, The Netherlands | a.tami@med.umcg.nl |
| Tjibbe Donker | National Institute for Public Health and the Environment, Bilthoven, The Netherlands | tjibbe.donker@rivm.nl |
| Helmut Mittermayer | National Reference Centre for Nosocomial Infections and Antimicrobial Resistance, Linz, Austria | helmut.mittermayer@elisabethinen.or.at |
| Karina Krziwanek | National Reference Centre for Nosocomial Infections and Antimicrobial Resistance, Linz, Austria | karina.krziwanek@elisabethinen.or.at |
| Sabine Stumvoll | National Reference Centre for Nosocomial Infections and Antimicrobial Resistance, Linz, Austria | sabine.stumvoll@elisabethinen.or.at |
| Walter Koller | Klinisches Institut fuer Hygiene und Medizinische Mikrobiologie, Vienna, Austria | walter.koller@meduniwien.ac.at, |
| Olivier Denis | Department of Microbiology, Université Libre de Bruxelles- Hopital Erasme, Belgium | odenis@ulb.ac.be |
| Marc Struelens | Department of Microbiology, Université Libre de Bruxelles- Hopital Erasme, Belgium | marc.struelens@ulb.ac.be, |
| Dimitr Nashev | National Center of Infectious and Parasitic Diseases, Sofia, Bulgaria | nashev@hotmail.com |
| Ana Budimir | Dept of Clinical and Molecular Microbiology, Clinical Hospital Centre Zagreb, Croatia | abudimir@kbc-zagreb.hr |
| Smilja Kalenic | Dept of Clinical and Molecular Microbiology, Clinical Hospital Centre Zagreb, Croatia | skalenic@mef.hr |
| Despo Pieridou-Bagatzouni | Department of Microbiology, Nicosia General Hospital, Cyprus | dbagatzouni@mphs.moh.gov.cy |
| Vladislav Jakubu | National Institute for Public Health, Prague, Czech Republic | vjakubu.cz |
| Helena Zemlickova | National Institute for Public Health, Prague, Czech Republic | hzemlickova@szu.cz |
| Henrik Westh | Hvidovre Hospital, Hvidovre, Denmark | Henrik.Westh@hvh.regionh.dk |
| Marit Sørum | Staphylococcal laboratory, Statens Serum Institut, Copenhagen, Denmark | mrr@ssi.dk |
| Robert Skov | Staphylococcal laboratory, Statens Serum Institut, Copenhagen, Denmark | rsk@ssi.dk |
| Frederic Laurent | INSERM, U851, French National Reference Centre for Staphylococci, Hospices Civils de Lyon, France | FREDERIC.LAURENT@recherche.univ-lyon1.fr |
| Jerome Ettienne | INSERM, U851, French National Reference Centre for Staphylococci, Hospices Civils de Lyon, France | JETIENNE@UNIV-LYON1.FR |
| Birgit Strommenger | Robert Koch Institut Bereich Wernigerode, Germany | Strommengerb@rki.de |
| Wolfgang Witte | Robert Koch Institut Bereich Wernigerode, Germany | WitteW@rki.de |
| Sofia Vourli | Department of Microbiology, National School of Pubic Health, Athens, Greece | svourli@freemail.gr |
| Alkis Vatopoulos | Department of Microbiology, National School of Pubic Health, Athens, Greece | avatopou@nsph.gr, micro@nsph.gr |
| Anni Vainio | National Institute for Health and Welfare, Helsinki, Finland | anni.vainio@thl.fi |
| Jaana Vuopio-Varkila | National Institute for Health and Welfare, Helsinki, Finland | jaana.vuopio@thl.fi |
| Miklos Fuzi | Semmelweis University, Budapest, Hungary | miklosfuzi@gmail.com |
| Erika Ungvári | National Center for Epidemiology, Budapest, Hungary | ungvari.erika@oek.antsz.hu |
| Stephan Murchan | Health Protection Surveillance Centre, Dublin, Ireland | stephen.murchan@hse.ie |
| Angela Rossney | National MRSA Reference Laboratory, St. James's Hospital, Dublin, Ireland | arossney@stjames.ie, |
| Edvins Miklasevics | Pauls Stradins Clinical University Hospital, Riga, Latvia | edvins.miklasevics@stradini.lv |
| Arta Balode | Pauls Stradins Clinical University Hospital, Riga, Latvia | arta.balode@stradini.lv,  31 |
| Gunnsteinn Haraldsson | Dept. of Clinical Microbiology, Landspitali University Hospital and University of Iceland, Reykjavik, Iceland | gah@hi.is |
| Karl G. Kristinsson | Dept. of Clinical Microbiology, Landspitali University Hospital and University of Iceland, Reykjavik, Iceland | karl@rsp.is |
| Monica Monaco | Department of Infectious, Parasitic and Immune-mediated Diseases, Istituto Superiore di Sanità, Rome, Italy | monica.monaco@iss.it, |
| Analisa Pantosti | Department of Infectious, Parasitic and Immune-mediated Diseases, Istituto Superiore di Sanità, Rome, Italy | pantosti@iss.it, |
| Michael Borg | Department of Infection Control, Mater Dei Hospital, Valetta, Malta | michael.a.borg@gov.mt, |
| Marga van Santen-Verheuvel | National Institute for Public Health and the Environment, Bilthoven, The Netherlands | Marga.van.Santen@rivm.nl |
| Xander Huijsdens | National Institute for Public Health and the Environment, Bilthoven, The Netherlands | Xander.Huijsdens@rivm.nl, |
| Lillian Marstein | MRSA Reference Laboratory in Norway, St. Olavs Hospital, University Hospital Trondheim, Norway | lillian.marstein@stolav.no |
| Trond Jacobsen | MRSA Reference Laboratory in Norway, St. Olavs Hospital, University Hospital Trondheim, Norway | trond.jacobsen@stolav.no |
| Gunnar Skov Simonsen | Department of Microbiology and Infection Control, University Hospital of North Norway, Tromsø, Norway | Gunnar.Skov.Simonsen@unn.no, |
| Marta Aires-de-Sousa | Laboratory of Molecular Genetics, Instituto de Tecnologia Quimica e Biologica, Lisbon, Portugal | msousa@esscvp.eu |
|  | Current address: Escola Superior de Saude da Cruz Vermelha Portuguesa, Lisbon, Portugal |  |
| Herminia de Lencastre | (1) Laboratory of Molecular Genetics, Instituto de Tecnologia Quimica e Biologica, Lisbon, Portugal, | (1) hml@itqb.unl.pt, |
|  | (2) The Rockefeller University, New York, USA | (2) lencash@mail.rockefeller.edu, |
| Agnieszka Luczak-Kadlubowska | Centre of Quality Control in Microbiology, Warsaw, Poland | luczakowa@cls.edu.pl |
| Waleria Hryniewicz | (1) National Medicines Institute, Warsaw, Poland | waleria@cls.edu.pl |
|  | (2) Centre of Quality Control in Microbiology, Warsaw, Poland |  |
| Monica Straut | National Institute of Research and Development for Microbiology and Immunology, Budapest, Romania | mstraut@cantacuzino.ro |
| Irina Codita | National Institute of Research and Development for Microbiology and Immunology, Budapest, Romania | icodita@cantacuzino.ro |
| Maria Perez-Vazquez | Centro Nacional de Microbiologia, Instituto de Salud Carlos III, Madrid, Spain | mperez@isciii.es |
| Oscar Cuevas | Centro Nacional de Microbiologia, Instituto de Salud Carlos III, Madrid, Spain | oscar.cuevas@isciii.es |
| Vesna Cvitkovic Spik | Institute of Microbiology and Immunology, Medical faculty, University of Ljubljana, Slovenia | vesna.cvitkovic-spik@mf.uni-lj.si |
| Manica Mueller-Premru | Institute of Microbiology and Immunology, Medical faculty, University of Ljubljana, Slovenia | MANICA.MUELLER-PREMRU@MF.UNI-LJ.SI |
| Sara Haegman | Swedish Institute for Infectious Disease Control, Stokholm, Sweden | sara.haeggman@smi.ki.se |
| Barbro Olsen- Liljequist | Swedish Institute for Infectious Disease Control, Stokholm, Sweden | barbro.liljequist@smi.ki.se |
| Matthew Ellington | Centre for Infections, Health Protection Agency, London, UK | matthew.ellington@hpa.org.uk |
| Angela Kearns | Centre for Infections, Health Protection Agency, London, UK | angela.kearns@hpa.org.uk |
| Robin Köck | Institute of Hygiene, University Hospital Münster, Germany | robin.koeck@ukmuenster.de |
| Alexander Mellmann | Institute of Hygiene, University Hospital Münster, Germany | alexander.mellmann@ukmuenster.de |
| Karsten Becker | Institute of Medical Microbiology, University **H**ospital Münster, Germany | kbecker@uni-muenster.de |
| Ulrich Vogel | Institute fuer Hygiene und Mikrobiologie der Universitaet Wuerzburg, Germany | uvogel@hygiene.uni-wuerzburg.de |
| Brian G. Spratt | Department of Infectious Disease Epidemiology, Imperial College, London, UK | B.Spratt@imperial.ac.uk |
| Dag Harmsen | Department of Periodontology, University Hospital Münster, Germany | dharmsen@uni-muenster.de, |
| Alexander W. Friedrich | Institute of Hygiene, University Hospital Münster, Germany | Alexander.Friedrich@ukmuenster.de |
