# Supplementary material for: Geographic Distribution of Staphylococcus aureus Causing Invasive Infections in Europe: A Molecular-Epidemiological Analysis
Source: PLoS Med. 2010 Jan 12;7(1):e1000215. doi: 10.1371/journal.pmed.1000215 (PMC2796391; doi:10.1371/journal.pmed.1000215)
Supplement: Text S2 — Author contributions of the Staphylococcus aureus Reference Laboratory Working Group members. (0.13 MB DOC) [file pmed.1000215.s002.doc]

**Text S2. Author Contributions of the European Staphylococcal Reference Laboratory Working Group**

| Hajo Grundmann, | conceived and designed the study, supervised the data analysis, wrote the manuscript |
| --- | --- |
| David M Aanensen, | developed the public domain web-based interactive mapping tool |
| Cees C. van den Wijngaard, | carried out spatial scan statistics |
| Artur J. Sabat, | received the crude data, kept communication with SRLs, initial data cleaning and integration |
| Jan Muilwijk, | carried out statistical analysis |
| Jos Monen, | reconciled SRL data with EARSS database, data management |
| Adriana Tami, | prepared presentation, illustrations, figures and tables |
| Tjibbe Donker | responsible for geographical information system |
| Helmut Mittermayer | responsible for national data collection and molecular typing |
| Karina Krziwanek | responsible for national data collection and molecular typing |
| Sabine Stumvoll | responsible for national data collection and molecular typing |
| Walter Koller | responsible for national data collection and molecular typing |
| Olivier Denis | responsible for national data collection and molecular typing |
| Marc Struelens | responsible for national data collection and molecular typing |
| Dimitr Nashev | responsible for national data collection and molecular typing |
| Ana Budimir | responsible for national data collection and molecular typing |
| Smilja Kalenic | responsible for national data collection and molecular typing |
| Despo Pieridou-Bagatzouni | responsible for national data collection and molecular typing |
| Vladislav Jakubu | responsible for national data collection and molecular typing |
| Helena Zemlickova | responsible for national data collection and molecular typing |
| Henrik Westh | responsible for national data collection and molecular typing |
| Marit Sørum | responsible for national data collection and molecular typing |
| Robert Skov | responsible for national data collection and molecular typing |
| Frederic Laurent | responsible for national data collection and molecular typing |
| Jerome Ettienne | responsible for national data collection and molecular typing |
| Birgit Strommenger | responsible for national data collection and molecular typing |
| Wolfgang Witte | responsible for national data collection and molecular typing |
| Sofia Vourli | responsible for national data collection and molecular typing |
| Alkis Vatopoulos | responsible for national data collection and molecular typing |
| Anni Vainio | responsible for national data collection and molecular typing |
| Jaana Vuopio-Varkila | responsible for national data collection and molecular typing |
| Miklos Fuzi | responsible for national data collection and molecular typing |
| Erika Ungvári | responsible for national data collection and molecular typing |
| Stephan Murchan | responsible for national data collection and molecular typing |
| Angela Rossney | responsible for national data collection and molecular typing |
| Edvins Miklasevics | responsible for national data collection and molecular typing |
| Arta Balode | responsible for national data collection and molecular typing |
| Gunnsteinn Haraldsson | responsible for national data collection and molecular typing |
| Karl G. Kristinsson | responsible for national data collection and molecular typing |
| Monica Monaco | responsible for national data collection and molecular typing |
| Analisa Pantosti | responsible for national data collection and molecular typing |
| Michael Borg | responsible for national data collection and molecular typing |
| Marga van Santen-Verheuvel | responsible for national data collection and molecular typing |
| Xander Huijsdens | responsible for national data collection and molecular typing |
| Lillian Marstein | responsible for national data collection and molecular typing |
| Trond Jacobsen | responsible for national data collection and molecular typing |
| Gunnar Skov Simonsen | responsible for national data collection and molecular typing |
| Marta Aires-de-Sousa | responsible for national data collection and molecular typing |
|  |  |
| Herminia de Lencastre | responsible for national data collection and molecular typing |
|  |  |
| Agnieszka Luczak-Kadlubowska | responsible for national data collection and molecular typing |
| Waleria Hryniewicz | responsible for national data collection and molecular typing |
|  |  |
| Monica Straut | responsible for national data collection and molecular typing |
| Irina Codita | responsible for national data collection and molecular typing |
| Maria Perez-Vazquez | responsible for national data collection and molecular typing |
| Oscar Cuevas | responsible for national data collection and molecular typing |
| Vesna Cvitkovic Spik | responsible for national data collection and molecular typing |
| Manica Mueller-Premru | responsible for national data collection and molecular typing |
| Sara Haegman | responsible for national data collection and molecular typing |
| Barbro Olsen- Liljequist | responsible for national data collection and molecular typing |
| Matthew Ellington | responsible for national data collection and molecular typing |
| Angela Kearns | responsible for national data collection and molecular typing |
| Robin Köck | complete analysis of new spa repeat results |
| Alexander Mellmann | performed the certification trial, evaluated spa typing results and the data of the BURP-analysis |
| Karsten Becker | contributed essentially to the spa typing capacity building and the international certification |
| Ulrich Vogel | developed the spa typing nomenclature |
| Brian G. Spratt | provided scientific advice, co-developed mapping tools, co-editied manuscript |
| Dag Harmsen | developed the spatyper software, co-organised the capacity building workshops, maintained and serviced the spaserver |
| Alexander W. Friedrich | maintained the SeqNet.org datbase, co-organised the capacity building workshops, responsible for proficiency testing |
